# The transcriptome assembly of the European freshwater mussel *Unio elongatulus* C. Pfeiffer, 1825

**DOI:** 10.1038/s41597-024-03226-y

**Published:** 2024-04-12

**Authors:** André Gomes-dos-Santos, Elza Fonseca, Nicoletta Riccardi, Mariana Hinzmann, Manuel Lopes-Lima, Elsa Froufe

**Affiliations:** 1 CIIMAR/CIMAR — Interdisciplinary Centre of Marine and Environmental Research, University of Porto, Terminal de Cruzeiros do Porto de Leixões, Avenida General Norton de Matos, S/N, P 4450-208, Matosinhos, Portugal; 2 CNR-Institute of Water Research, Verbania Pallanza, VialeTonolli, Italy; 3 BIOPOLIS Program in Genomics, Biodiversity and Ecosystems, CIBIO, Centro de Investigação em Biodiversidade e Recursos Genéticos, InBIO Laboratório Associado, Campus de Vairão, Universidade do Porto, Vairão, Portugal; 4 IUCN SSC Mollusc Specialist Group, c/o IUCN, Cambridge, UK

**Keywords:** Gene expression, Conservation biology

## Abstract

Freshwater mussels of the order Unionida are a global conservation concern. Species of this group are strictly freshwater, sessile, slow-growing animals and, extremely sensitive to environmental changes. Human-mediated changes in freshwater habitats are imposing enormous pressure on the survival of freshwater mussels. Although a few flagship species are protected in Europe, other highly imperilled species receive much less attention. Moreover, knowledge about biology, ecology, and evolution and proper conservation assessments of many European species are still sparse. This knowledge gap is further aggravated by the lack of genomic resources available, which are key tools for conservation. Here we present the transcriptome assembly of *Unio elongatulus* C. Pfeiffer, 1825, one of the least studied European freshwater mussels. Using the individual sequencing outputs from eight physiologically representative mussel tissues, we provide an annotated panel of tissue-specific Relative Gene Expression profiles. These resources are pivotal to studying the species’ biological and ecological features, as well as helping to understand its vulnerability to current and future threats.

## Background & Summary

Being the animal group with the highest extinction rate (6.3%) and one of the most imperilled taxa (~43% threatened species)^1^, freshwater mussels of the order Unionida have become a global conservation concern^2–5^. This order contains the most diverse group of strictly freshwater bivalves^6,7^, which share several biological traits that make them extremely vulnerable to ecological disturbance^2,4,5^. Most freshwater mussels have long life spans with slow growth rates, a sessile adult stage with limited vagility, and a highly specialised life cycle that includes an obligatory parasitic larval stage (glochidia) that must attach to freshwater fish for food and dispersal^8,9^. Throughout the many stages of their complex life cycle (e.g., sperm release to larval release), mussels are particularly vulnerable to external ecological factors (e.g., water and sediment quality and the number and diversity of host fish). Consequently, the stability of these ecological factors is key to the survival of freshwater mussels^2,8,10^. Freshwater habitats are among the most intensively modified ecosystems, with massive pressures on the reproduction and recruitment of freshwater mussels that cannot adapt to the pace of these changes^2,4,8,11–13^. Nearly 50% of freshwater mussel species are threatened in Europe (IUCN 2023). Steep population declines and local extinctions have been widely documented, with long-term surveys revealing no halt to these trends^2,14^. In addition, European conservation actions and funding have largely been absorbed by a few flagship species, with almost 33 million € invested in Life projects focused on the conservation of just three freshwater mussel species^15^. Conversely, other species that are highly imperilled or experiencing severe population declines receive much less attention^2,8,14^. Perhaps more worryingly, some European species still lack a comprehensive understanding of their evolutionary, biological, and ecological characteristics or even adequate conservation assessments. This is the case of the species *Unio elongatulus* C. Pfeiffer, 1825, whose taxonomy has been difficult to resolve until recently^2,16–18^.

**Fig. 1:**
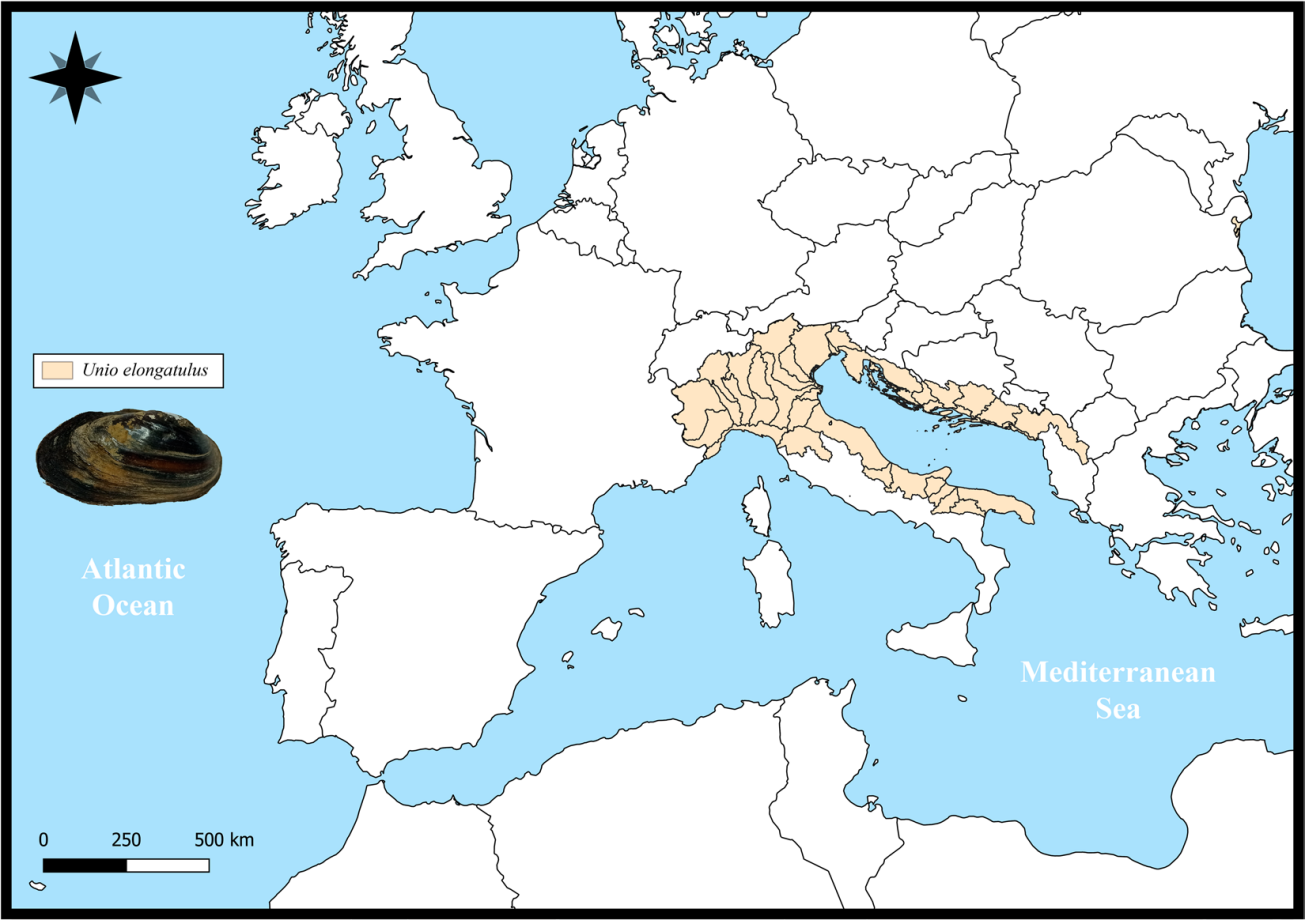
Maps of the *Unio elongatulus* distributions. The maps of the potential distributions were produced by overlapping points of recent presence records (obtained from Lopes-Lima *et al*.^2^) with the Hydrobasin level 6 polygons^72^.

At the end of the 20^th^ century, the species was considered to be threatened and has since been legally protected in EU countries (listed in the Bern Convention [Appendix III] and the Habitats Directive [Annex V]). However, the concept of *U. elongatulus* at the time of the introduction of these directives now includes three different species that are currently recognised as valid species, i.e. *U. elongatulus*, *Unio ravoisieri* Deshayes, 1848, and *Unio mancus* Lamarck, 1819. Due to this unstable taxonomy, until recently the distribution of the species was poorly defined and often misplaced^2,16,17^ and its conservation status was unclear and therefore not assessed by the IUCN. With the advent of molecular tools, *U. elongatulus* has been separated from *U. mancus* and *U. ravoisieri* and its distribution is now restricted to rivers and lakes draining into the Adriatic Sea in Italy (from the Ofanto River in the South to the Po River in the North Adriatic) and the Balkans (Croatia and Albania), as well as some Tyrrhenian Sea drainages in Northern Italy (e.g. Arno and Serchio) (Fig. 1)^16–18^. Furthermore, due to the recent redefinition of the species, most aspects of its biology and ecology are equally understudied, hindering a full understanding of its vulnerability and the anticipation and/or planning of conservation measures^2,16,17^.

When applied to the study of non-model organisms, genomic methods provide highly informative tools to enhance the success of conservation efforts^19–22^. Genomic approaches provide robust inferences about genetic diversity, population structure and connectivity and selective and adaptive traits, which in turn allow the identification of significant conservation units, traits and genetic elements and provide a comprehensive picture of population health^19–25^. In addition, genomic tools significantly increase the accuracy of predicting the impact of anthropogenic threats and climate change on natural populations^21,23–25^. Nevertheless, the availability of genomic resources is still scarce for many non-model organisms^26^. This is the case for freshwater mussels with nearly a thousand known species, for which only six whole genome assemblies^27–34^ and fewer than 20 transcriptomes^35–48^ are available. The generation of novel genomic resources for the study of freshwater mussels is therefore of fundamental importance. While genome assemblies are undoubtedly the most powerful tools for understanding the biological and evolutionary traits of species, functionally informative methods such as transcriptomics are also important to provide information on gene structure and expression profiles^49^. This is particularly significant for poorly known species, as these resources represent easily accessible and affordable approaches to accessing highly informative biological, ecological, and evolutionary genetic traits. Freshwater mussels transcriptomics has been instrumental in investigating responses to thermal stress, water depletion, pollution, and bacterial infection (e.g^35,37,39–41^), traits that are intrinsically linked to their global conservation.

Here we present a highly comprehensive multi-tissue transcriptome assembly for one of the least studied freshwater mussels in Europe, *U. elongatulus*. In addition, we provide a well-annotated panel of tissue-specific relative gene expression profiles, using the individual sequencing outputs of the eight functionally representative tissues, i.e., adductor muscle, foot, gill, gonad, gut, hepatopancreas, mantle and palps. Altogether, these resources will provide a key tool to explore and decipher many biological and ecological aspects of this freshwater mussel species, which in turn will help to understand its vulnerability to current and future threats and shed light on the evolutionary patterns of European freshwater mussels.

## Methods

### Animal sampling

One individual of *U. elongatulus* was collected from Lake Maggiore in Italy (Table 1) and transported alive to the laboratory, where the tissues (i.e., adductor muscle, foot, gill, gonad, gut, hepatopancreas, mantle and palps) were immediately dissected, flash frozen and stored at −80 °C, at the CIIMAR tissue and mussels collection under voucher code BIV10776. The shell was also deposited at CIIMAR under the same voucher code.

**Table 1:** MixS descriptors for the *Unio elongatulus* specimen.

Sample	*Unio elongatulus*
Investigation_type	Eukaryote
Project_name	Transcriptome assembly of the freshwater musssles’ european species *Unio elongatulus*
Lat_lon	
Geo_loc_name	Italy
Collection_date	
Env_package	Water
Seq_meth	Illumina HiSeq 4000
Assembly method	Trinity
Collector	
Sex	Undetermined
Maturity	Mature

### RNA extraction, library construction, and sequencing

Total RNA of each tissue was extracted with the NZY Total RNA Isolation Kit (NZYTech, Lda. - Genes and Enzymes), according to the manufacturer’s guidelines. A DS-11 Series spectrophotometer/fluorometer was used to estimate RNA concentration (ng/μl) and absorbance measurements (OD260/280 ratio values) (adductor muscle 154.88 ng/μl, foot 824.48 ng/μl, gill 742.08 ng/μl, gonad 1234.08 ng/μl, gut 354.08 ng/μl, hepatopancreas 2993.28 ng/μl, mantle 441.28 ng/μl and palps 342.08 ng/μl). RNA extractions from the eight tissues were sent to Macrogen, Inc., where strand-specific libraries were built (insert size of 250–300 bp) and total RNA was sequenced using 150 bp paired-end reads on the Illumina HiSeq. 4000 platform.

### Read processing and *de novo* transcriptome assembly

Raw sequencing reads for each tissue were inspected with FastQC (version 0.11.8) software (http://www.bioinformatics.babraham.ac.uk/projects/fastqc/), filtered for Illumina adaptors and quality using Trimmomatic (version 0.38)^50^ (LEADING:5 TRAILING:5 SLIDINGWINDOW:5:20 MINLEN:36) and corrected for random sequencing errors using a Rcorrector (version 1.0.3)^51^ (default parameters). The trimmed and corrected reads were concatenated and used for *de novo* assembly of the entire transcriptome using Trinity (version 2.13.2)^52,53^ (default parameters). The resulting assembly was screened for putative contaminations by blast search against the Univec (Download; 02/04/2019) and NCBI-nt (Download; 24/08/2021)^54^ nucleotide databases using Blast-n (version 2.11.0)^55^. Assembled transcripts matching the phylum Mollusca (NCBI: taxid 6447) or with no match at all were retained. Conversely, transcripts matching other taxa with over 100 bp alignment length, an e-value of 1e-5 or lower, and over 90% of identity score, were flagged as contaminants and excluded. Moreover, all transcripts with any match to the Univec database were also excluded from the assembly.

### Redundancy removal

Redundancy from the filtered assembly was removed using the hierarchical contig clustering method applied by Corset (version 1.0.9)^56^. Briefly, quality treated reads for each tissue were mapped to the transcriptome assembly using Bowtie2 (version 2.3.5) (parameter: --no-mixed --no-discordant --end-to --end --all --score-min L, −0.1, −0.1) and after Corset used to exclude redundancies and transcripts with fewer than 10 reads mapped. The general characteristics, structural integrity, and completeness of the transcriptome (before and after Corset) were assessed using TransRate (version 1.0.3)^57^ and Benchmarking Universal Single-Copy Orthologs tool (BUSCO version 3.0.2). For BUSCO, completeness estimations were performed using the lineage-specific libraries available for Eukaryota and Metazoa^58^.

### Open reading frame prediction and transcriptome annotation

Open reading frames (ORFs) prediction was performed using Transdecoder (version 5.3.0) (https://transdecoder.github.io/), homology and protein searches being performed in the PFAM^59^ and UniProtKB/Swiss-Prot^60^ databases^59^ using the Blast-p (version 2.12.0)^55^ and hmmscan of hmmer2 (version 2.4i)^61^, respectively. Subsequently, a structural annotation file was produced using Gtf/Gff Analysis Toolkit (AGAT) (version 0.8.0)^62^, supplying the software with the Transdecoder gff output file and the fasta transcriptome assembly. This allowed the generation of protein, transcript, cds and mrdn fasta files with standardised feature names. Functional annotation was performed using InterProScan (version 5.44.80) and Blast-n/p/x searches against several databases. Protein sequences were queried against the NCBI protein databases NCBI-RefSeq Database (Download; 10/03/2022)^63^, NCBI-nr – non-redundant database (Download; 15/12/2021)^54^ and InterPro database (Download; 30/03/2019), using the Blast-p/x tools from DIAMOND (version version 2.0.13)^64^. Transcripts were queried against the NCBI-nt and NCBI-nr databases using the Blast-n tool of NCBI and the Blast-x tool from DIAMOND. All blast (outfmt6 files) and the InterProScan (tsv file) outputs were combined into the gff3 annotation file using AGAT. Gene names were assigned, per sequence, based on the best blast hit (Gene symbol – NCBI Accession Number) and following the hierarchical order: 1- Blast-p Hit in RefSeq database; 2 - Blast-p Hit in NCBI-nr database; 3 - Blast-x Hit in NCBI-nr database; 4 - Blast-n Hit in NCBI-nt database.

### Read alignment and tissues specific relative gene expression analysis

For each of the individual sequencing output, Relative Gene Expression (RGE) analyses were conducted using the tool “align_and_estimate_abundance.pl”, implemented through Trinity, specifying “RSEM” as the estimation method and “Bowtie2” as an aligner, using the non-redundant assembly as a reference (Parameters: --est_method RSEM --aln_method bowtie2 --trinity_mode).

## Data Records

The sequencing outputs and transcriptome assembly were deposited in NCBI under the BioProject accession number PRJNA1030315: BioSample accession SAMN37904383; Transcriptome Shotgun Assembly (TSA) accession GKPW00000000^65^; Sequence Read Archive (SRA) accessions SRR26451330, SRR26451329, SRR26451328, SRR26451327, SRR26451325, SRR26451324, SRR26451323, SRR26451326^66^. The remaining information was uploaded to figshare^67^. In detail, the files uploaded to figshare include, the filtered trinity redundant assemblies (Uel_trinity_filtered.fasta), the non-redundant transcriptomes (Uel_transcriptome.fa), transcript files (Uel_genes.fa), messenger RNA file (Uel_mrna.fa), open reading frames predictions (Uel_cds.fa), open reading frames proteins predictions (Uel_proteins.fa) as well as the annotation files (Uel_Annotation_sorted.gff3.gz) and the Relative Gene Expression Count tables for all tissues (*_RSEM.isoforms.results).

## Technical Validation

### Raw datasets and pre-assembly processing quality control

**Fig. 2:**
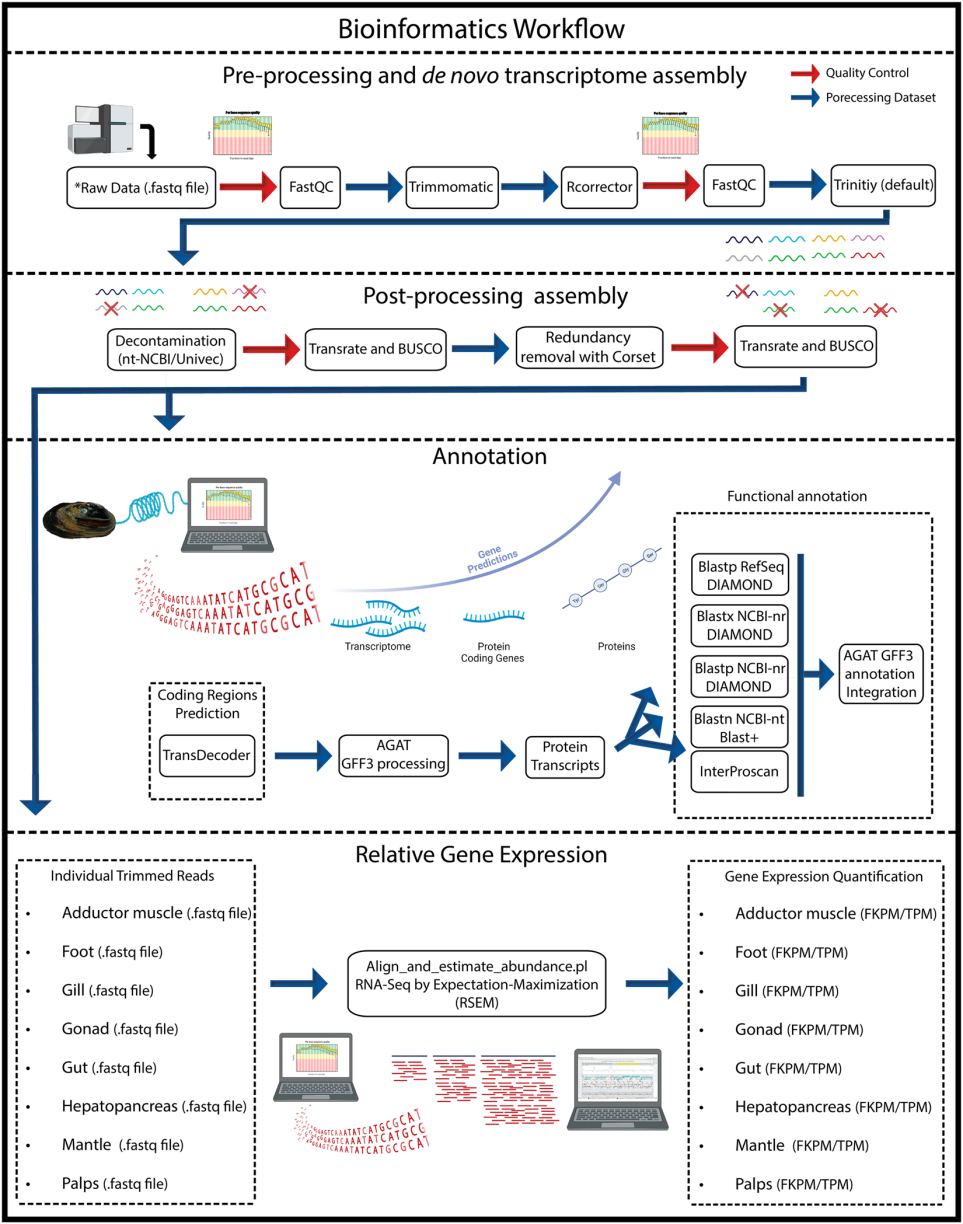
Bioinformatics pipeline applied for the transcriptome assembly and annotation. Auxiliary representative figures were created with BioRender.com.

Overall, the raw sequencing output for each tissue was evenly distributed, resulting in almost 22 million reads (M) for adductor muscle, 20 M for foot, 23 M for gill, 23 M for gonad, 19 M for gut, 23 M for hepatopancreas, 22 M for mantle, and 22 M for palps. The overall quality of the raw data was substantially good as demonstrated by the fastqc report (Fig. 2), thus resulting in a low number of low-quality reads being removed during quality trimming (Trimmomatic), less than 2% for each tissue dataset (Table 2). Nevertheless, trimming and error correction (Rcorrector) improved the quality of the reads (Fig. 2).

**Table 2:** Basic statistics of raw sequencing datasets and percentages of removed reads after the preassembly processing strategy.

Raw Reads	Adductor muscle	Foot	Gill	Gonad	Gut	Hepatopancreas	Mantle	Palps
Raw sequencing reads	21,778,666	20,378,331	23,201,882	23,287,603	19,099,385	23,135,183	22,093,607	22,619,225
Trimmomatic reads removed	292,882 (1.34%)	289,539 (1.42%)	319,323 (1.38%)	319,966 (1.37%)	250,782 (1.31%)	287,759 (1.24%)	347,166 (1.57%)	300,015 (1.33%)
Reads used in assembly	21,485,784	20,088,792	22,882,559	22,967,637	18,848,603	22,847,424	21,746,441	22,319,210

### Transcriptome assembly metrics

The Trinity transcriptome assembler, used with default parameters, is a very fast and effective approach for unionid transcriptome assemblies^35,38–41,48^. This is demonstrated again in our multi-tissue *de novo* transcriptome (Table 3). The quality of the Trinity output, even before any redundancy removal, is evidenced by the Transrate statistical report, with an N50 length of 1,035 in a total of 1,950,805 transcripts, as well as by the completeness report generated using BUSCO, which reported 100% and 99.2% total genes found (complete + fragmented) for the lineage-specific libraries available for Eukaryota and Metazoa, respectively (Table 3). These values are within the range reported for other Unionida transcriptome assembly projects^35–39,41,43–45,47^, including other congeneric European species^48^.

**Table 3:** Transrate and Busco scores of redundant transcriptome assembly, before and after redundancy removal.

Basic Statistics	Total Transcriptome	Non redundant Transcriptome
Number of transcripts	1,950,805	400,748
n bases	1,401,701,543	479,357,670
Mean transcript lenght (bp)	718.490	1196.156
Number of transcripts over 1 K nt	348,952	177,728
Number of transcripts over 10 K	1,268	387
N90 trancript lenght (bp)	305	605
N70 trancript lenght (bp)	561	1,027
N50 trancript lenght (bp)	1,035	1,496
N30 trancript lenght (bp)	1,898	2,248
N10 trancript lenght (bp)	4,102	4,223
Percentage of GC (%)	0.354	0.349
Busco analysis (%)		
BUSCO Complete (Single + Duplicated)	100/99.2	90.8/91.7
BUSCO Single*	39.9/40.2	88.8/89.3
BUSCO Duplicated*	60.1/59	2/2.4
BUSCO Fragmented*	0/0.6	5/4.7
BUSCO Missing*	0/0.2	4.2/3.6
Total Buscos Found*	100/99.8	95.8/96.4

*euk/met. Euk: Dataset with 303 genes of Eukaryota library profile. Met: Dataset with 978 genes of Metazoa library profile.

### Post-assembly processing and annotation verification

Before proceeding with transcript redundancy removal, the assembly was screened and cleaned of putative contaminations, using an approach developed by the team and previously successfully tested on freshwater mussels’ transcriptomes^48,68,69^. This approach identifies sequences based on homology and only excludes transcripts with well-supported matches between different databases (i.e., NCBI-nt and Univec), thus avoiding the exclusion of unambiguous matches. Using this approach, a total of 5,758 were identified as likely contaminations and removed from the assembly, which was then used for redundancy removal using Bowtie2/Corset. Bowtie2 mapping percentages per tissue were as follows: 89.30% for adductor muscle, 89.22% for foot, 87.46% for gill, 87.69% for gonad, 84.20% for gut, 89.21% for hepatopancreas, 85.87% for mantle, and 87.48% for palps. Corset uses a hierarchical clustering of contig sharing read alignments, resulting in unbiased redundancy exclusion of total transcripts, rather than removing the largest isoform based on protein-coding transcripts only^56^ and has been successfully applied by the team to freshwater mussel transcriptome assemblies^48^. Corset was highly efficient in redundancy removal, removing almost 80% of the total number of transcripts and reducing the reported BUSCO’s duplicated genes from around 60% to 2%, with a residual impact on the number of total genes found (both above 90%) (Table 3). These results, reinforce the importance of removing redundancy after Trinity, which despite producing highly complete assemblies, also produces many duplicated and/or low-read-supported transcripts. In the end, Corset significantly reduced the initial assembly, while maintaining its completeness and removing putative errors introduced during the assembly. Open reading frames (ORF) prediction was performed on the decontaminated non-redundant assembly using TransDecoder, resulting in a total of 63,067 ORF (Table 4). Functional annotation was performed on all the predicted amino acid sequences using a well-established pipeline within the team^27,33,48,70,71^, and the results are presented in Table 5, which shows the detailed hit list with several different databases used for annotation. In total, 30,605 and 34,958 transcripts were functionally annotated by InterProScan and Blast search, respectively (Table 5). These values are very similar to the number of functionally annotated genes reported for the genome assemblies of two congeneric European species, i.e., *Unio delphinus* (32,089)^70^ and *Unio pictorum* (34,137)^33^, fitting well within the values reported for other Unionida genome assemblies^27–30,34,71^. Conversely, the numbers reported here are slightly higher than those reported in the recently published transcriptomes of four *Unio* species, i.e., *Unio pictorum* (InterProScan:14,723; Blast:24,194), *Unio mancus* (InterProScan:14,971; Blast:24,775), *Unio crassus* (InterProScan:20,432; Blast:51,937) and *Unio delphinus* (InterProScan:20,637; Blast:32,688)^48^. However, the transcriptome assemblies were only based on a single tissue (i.e., gill), which probably explains the reduced number of annotated transcripts. Overall, these results confirm the global quality and completeness of the transcriptome assembly presented here and highlight the importance of using a multi-tissue approach for transcriptome assemblies in freshwater mussels to ensure the obstinances of a representative panel of transcripts. Finally, we used the sequencing outputs for each sample tissue to generate and provide the tissue-specific relative gene expression profiles. The full RGE tables are available in FigShare, where the quantification of gene expression is displayed using both Fragments Per Kilobase of transcript per Million (FKPM) and Transcripts Per Million (TPM) values (Gene IDs according to the gff annotation file). The number of genes with TPM and FKPM values > 1 identified by the two methods and for each tissue is presented in Table 5.

**Table 4:** Functional annotation statistics for the final transcriptome assembly.

Structural annotation	*Unio elongatulus*
Number of transcripts	400,748
Number of cdss	63,067
Number of exons	63,067
Total gene length	479,357,670
Total cds length	49,250,289
Total exon length	121,261,967
mean gene length	1,196
mean cds length	780
mean exon length	1,922
**Functional annotation Blast**	***Unio elongatulus***
Blast-p/x/n hits (NCBI-RefSeq; NCBI-nr; NCBI-nt)	34,958
**Functional annotation InterPro**	***Unio elongatulus***
CDD	8,363
Coils	5,690
GO	13,282
Gene3D	19,121
Hamap	261
InterPro	23,857
KEGG	1,028
MetaCyc	966
MobiDBLite	11,177
PIRSF	737
PRINTS	3,637
Pfam	19,773
ProSitePatterns	4,760
ProSiteProfiles	11,609
Reactome	4,239
SFLD	79
SMART	9,054
SUPERFAMILY	19,110
TIGRFAM	847
**Total**	30,605

**Table 5:** Relative Gene Expression counts above 1 for each tissue in Fragments Per Kilobase of transcript per Million (FKPM) and Transcripts Per Million (TPM), for genes with TPM and FKPM.

Tissue	Adductor	Foot	Gill	Gonad	Gut	Hepatopan	Mantle	Palps
Total FPKM Ove 1	129007	135286	134851	104523	137531	109797	136938	147194
Total TPM Ove 1	123266	126316	118298	107559	124259	100006	125152	129729

The present results provide an important genomic resource that will serve as an essential tool for future studies investigating the biology of one of the least studied freshwater mussel species in Europe, as well as contributing to the broader understanding of the biology and evolution of freshwater mussels as a whole.

## Data Availability

All software with respective versions and parameters used to generate the resources presented here (i.e., transcriptome assembly, pre- and post-assembly processing stages, and transcriptome annotation) are listed in the Methods section. Software programs without associated parameters were used with the default settings.
